# Copper infused fabric attenuates inflammation in macrophages

**DOI:** 10.1371/journal.pone.0287741

**Published:** 2023-09-15

**Authors:** Safoura Zangiabadi, Khalil P. Chamoun, Khang Nguyen, Yitian Tang, Gary Sweeney, Ali A. Abdul-Sater

**Affiliations:** 1 School of Kinesiology and Health Science, Muscle Health Research Centre (MHRC), York University, Toronto, Canada; 2 Department of Biology, York University, Toronto, Ontario, Canada; Wayne State University School of Medicine, UNITED STATES

## Abstract

While inflammation is an important immune response for protection from infections, excessive or prolonged inflammation can lead to a variety of debilitating diseases including skin disease, diabetes, heart disease, stroke, autoimmune diseases and cancer. Inflammation is a graded response that is typically initiated when resident macrophages sense the presence of pathogens or damage in the tissue and produce inflammatory cytokines and chemokines to kill the pathogen, clear debris and dead tissue, and initiate tissue repair. Here we show that copper-infused fabrics can prevent inflammation by blocking the production of inflammatory cytokines from macrophages after being exposed to LPS, a component of bacterial cell wall. Mechanistically, we show that copper-infused fabrics can significantly reduce the NF-κB and IRF3 activation in LPS-stimulated macrophages. Given the importance of excessive inflammation in diabetes, we show that copper can reduce insulin resistance mediated by inflammatory cytokines in muscle cells. Our data show that copper infused fabrics may be useful to reduce excessive inflammation in macrophages and improve insulin sensitivity in skeletal muscles.

## Introduction

Inflammation is a rapid and essential immune response that is initiated in response to tissue damage and injury, whether that is due to infections or sterile insults [1, 2]. Localized inflammation causes vasodilation, increased vascular permeability, which allows immune cells to infiltrate the injury site. This is accompanied by edema, heat and swelling. The end result is clearing the infection, removing dead tissues, and initiating tissue repair [2, 3].

Tissue resident macrophages are usually the first cells that respond to the presence of a foreign threat or sterile injury and initiate the inflammatory response. Macrophages employ families of germline-encoded pattern recognition receptors (PRRs) that exhibit broad host specificity towards a variety of pathogen-associated molecular patterns (PAMPs) [4, 5].

PRRs include among others Toll-like receptors (TLRs), which upon recognizing PAMPs trigger the activation of transcription factors (e.g. nuclear factor κB (NF-κB), mitogen activated protein kinases (MAPKs) and interferon regulatory factor 3 (IRF3) to direct the expression and secretion of a number of pro-inflammatory cytokines (e.g., tumor necrosis factor alpha (TNF-α), interleukin 1 (IL-1), IL-6 and antiviral cytokines, called type-I Interferons (e.g. IFN-α, IFN,β). Moreover, anti-inflammatory cytokines (e.g. IL-10, TGF-β) are produced to reduce inflammation and initiate tissue repair [6–8].

Despite the importance of the inflammatory response in healing the damaged tissue, unnecessary or prolonged/chronic inflammation can be detrimental and cause excessive tissue damage that has been associated with a variety of human diseases. These include autoimmune diseases (e.g. rheumatoid arthritis, dermatitis, lupus, etc…), cardiovascular diseases, stroke, type 2 diabetes and cancer. Therefore, developing anti-inflammatory products, including pharmaceutical agents and other alternatives, has been on the rise [9–11].

The anti-microbial properties of copper and its alloys, including brass and bronze, have long been recognized and used in different applications even before the concept of microbes was discovered [12, 13]. Upon exposure to copper or copper-containing alloy surfaces, pathogenic bacteria are rapidly killed in a process called “contact killing” [13]. The release of copper ions from the copper surface is the key event in contact killing [14]. Indeed, the use of copper touch surfaces to reduce the microbial load on contact surfaces in various healthcare settings led to renewed interest in investigating the mechanism of how copper exerts its anti-microbial effects. Recent studies have elucidated the mechanisms of copper-mediated contact killing that include damage of bacterial membrane, oxidative stress by production of reactive oxygen species (ROS), and DNA degradation [14, 15]. A study by Hong et al. 2012 examined the mechanism of contact-mediated killing of E. coli when exposed to different copper alloy surfaces containing 70%-99% Cu or in a medium containing CuSO4. They demonstrated that nonenzymatic oxidative damage of the E.coli membrane phospholipids resulted in the loss of membrane integrity and cell death [16].

Early civilizations used copper to sterilize drinking water, disinfect wounds, and treat various infections [17]. Recently, anecdotal evidence suggested that incorporating copper into wearable accessories has health benefits and can promote an anti-inflammatory state to alleviate arthritis and other inflammatory based diseases [17, 18]. Although, some studies have shown anti-inflammatory effects of copper in animal models and in vitro, other studies have shown that copper can behave as an inflammatory agent and increase the levels of damaging free radicals (reviewed in [18–20]). In particular, exposure to copper oxide nanoparticles (CuONPs), which were used in antimicrobial textiles and other applications, was shown to cause adverse health effects. Studies in rodents demonstrated that oral exposure to CuONPs can induce oxidative stress and liver toxicity [21] as well as aggravate airway inflammation and the development of asthma [22]. Hence, new generation textiles were introduced with fabrics impregnated with copper ions or copper salts.

Here, we report that these copper-infused fabrics can attenuate pro-inflammatory cytokine gene expression and production from human macrophages (derived from THP-1 cell line) that have been exposed to bacterial lipopolysaccharide (LPS). Mechanistically, we show that media leachates obtained from these copper-infused fabrics inhibit the activation of the key transcription factors NF-κB and IRF3. Additionally, copper reduced insulin resistance mediated by pro-inflammatory cytokines in skeletal muscle cells. To our knowledge, this is the first report that demonstrates that copper-infused fabric have potent anti-inflammatory properties on a human macrophage cell line.

## Materials and methods

### Cell culture and reagents

THP-1 human monocytic leukaemia cells were obtained from Sigma-Aldrich (St Louis, MO). THP-1 cells were cultured in a humidified incubator at 37°C with 5% CO_2_ in RPMI 1640 (Sigma-Aldrich) supplemented with 10% heat inactivated Fetal Bovine Serum (Wisent Bio Products, Saint-Bruno-de-Montarville, QC), 2-Mercapthoethanol, GPPS, and non-essential amino acids (Sigma-Aldrich).

RAW-Dual cells (IRF-Lucia/KI-[MIP-2]SEAP) generated from RAW 264.7 murine macrophages were obtained from InvivoGen (San Diego, CA), and stably express two reporter genes encoding SEAP (secreted embryonic alkaline phosphatase) and Lucia luciferase, which report the activation of NF-κB and IRFs, respectively. Cells were cultured in Dulbecco’s modified Eagle’s medium (Sigma-Aldrich) supplemented with 10% heat inactivated FBS and cultured at 37°C in humidified 5% CO_2_.

### Media leachate preparation from copper-infused fabrics

100% Polyester 380 GSM (grams per square meter) fabrics that either contain no copper (control) or were infused with copper (Natuverex by Fine Cotton Factory; Toronto, ON) were cut into 10x10 cm square pieces and sterilized in an autoclave. Each fabric was placed separately in a 10 cm Petri dish containing 30 ml of growth media (RPMI 1640 with 10% FBS, NEAA, GPPS, and 2-ME). Leachates were prepared by incubating the fabrics in growth medium for 24 hours at 37°C with 5% CO_2_. The leachates were collected and used to culture differentiated THP-1 cells in a dose dependent manner; full leachate (100% leachate), a 1:1 ratio of leachate to normal growth media (50% leachate) or 1:3 ratio of leachate to normal growth media (25% leachate).

### Cell stimulation

THP-1 human monocytic cells were seeded at 5x10 ^5 and differentiated into macrophages following overnight incubation with 100 nM phorbol-myristate-acetate (PMA; Sigma-Aldrich). Then cells were cultured in either RPMI growth media supplemented with 10% FBS, 100% media leachates from copper infused fabrics (unless otherwise specified), or 20 μM of copper(II) chloride dihydrate (CuCl2; Sigma-Aldrich) for 24 hours. Cells were then treated with 100 ng/ml lipopolysaccharide (LPS from Escherichia coli O55:B5; Sigma-Aldrich) for the indicated times.

### Copper assay

Copper concentration in media leachates were measured using a copper assay kit (Sigma, Cat No. MAK127), as per manufacturer’s instructions.

### Preparation of cell extracts and immunoblotting

Differentiated THP-1 cells were scraped and lysed in 2% NP40 lysis buffer containing protease inhibitor cocktail (Roche, Basel, Switzerland), phosphatase inhibitor cocktail 3, Sigma-Aldrich) and Dithiothreitol (DTT, Sigma-Aldrich). Cell lysates were loaded onto a 10% SDS-polyacrylamide gel, electrophoresed, and transferred to a polyvinylidene difluoride membrane (Bio-Rad). Blots were blocked with 5% skim milk in TBST for 1 hour at room temperature. The membranes were probed for IκB-α (clone L35A5; Cat. No. 4814S; Cell Signaling, Danvers, MA), p-IκB-α (clone 14D4; Cat. No. 2859S; Cell Signaling), β-Actin (clone 937215; Cat. No. MAB8929; R&D Systems), and incubated overnight at 4°C. Subsequently, the membranes were incubated with peroxidase-conjugated secondary anti-mouse and anti-rabbit antibody (Jackson ImmunoResearch) for 1 hour at room temperature. Immunoreactive proteins were detected with Clarity^™^ ECL Western blotting detection reagent (Bio-Rad) using ChemiDoc MP imaging system (Bio-Rad).

For insulin signaling experiments, L6 rat skeletal muscle cells were grown in alpha‐minimum essential medium (AMEM) media containing 10% fetal bovine serum (FBS) and 1% antibiotic/antimycotic at 37°C with 95% air and 5% CO2. L6 cells were seeded on 6 well plates to 80–90% confluency. Cells were treated with conditioned media collected from THP-1 cells that were stimulated with LPS in the presence of control or copper leachates for 24 hours followed by 10 min-treatment of insulin 100 nM (Lilly; Humulin-R U-100). Cell lysates were run on 8% SDS-PAGE gels and probed with phospho-specific Akt antibody (p-Akt; Cell Signaling) or total Akt antibody (t-Akt; Cell Signaling; Cat. No. 9272). Western blot (WB) band intensity of p-Akt was quantified using ImageJ software, normalized to t-Akt antibody.

### RNA isolation and real-time PCR

mRNA was isolated from the THP-1 cells using the Aurum ^™^ Total RNA mini kit (Bio-Rad) following manufacturer’s instruction. Total RNA was converted to cDNA by standard reverse transcription with M-MuLV Reverse Transcriptase (New England Biolabs, Ipswich, MA) and oligo(dT) primers (Qiagen, Hilden, Germany), and then amplified with a Ssoadvanced SYBR Green master mix (Bio-Rad) and human primers (listed below) using a CFX384 Touch Real-Time PCR Detection System (Bio-Rad). The primers for human GAPDH were 5’- TGACAACAGCCTCAAGAT- 3’ forward and 5’ GAGTCCTTCCACGATACC- 3’ reverse. Primers for IL-10 were 5’-GCTGGAGGACTTTAAGGGTTACCT-3’ forward and 5’-CTTGATGTCTGGGTCTTGGTTCT-3’ reverse. Primers for IL-1β were 5’- TACATCAGCACCTCTCAAG-3’ forward and 5’- ATTCAGCACAGGACTCTC- 3’ reverse. Primers for IFN-β were 5’-TCAAGGACAGGATGAACTT-3’ forward and 5’-GACATTAGCCAGGAGGTT-3’ reverse. Primers for TNF were 5’-CTGACATCTGGAATCTGGA-3’ forward and 5’-GTCTCAAGGAAGTCTGGAA-3’ reverse. Primers for IL-6 were 5’-TGAGAGTAGTGAGGAACAAG-3’ forward and 5’-CGCAGAATGAGATGAGTTG-3’ reverse. Real time PCR included initial denaturation at 95°C for 10 min, followed by 40 cycles of 95°C for 30 s, 55°C for 1 min, 72°C for 1 min, and one cycle of 95°C for 1 min, 55°C for 30 s, 95°C for 30 s. Fold change in gene expression was calculated using the delta-delta Ct (2^–ΔΔCt^) method with respect to untreated (PBS; φ) controls. Data are averages from three biological replicates and variations reported as standard deviations.

### ELISA

Supernatants from cultured cells were used to assay protein concentration of IL-1β (Cat. No. 88-7261-88; Thermo Fisher Scientific) and CxCl1 (Groα; Cat. No. 88-52122-88; Thermo Fisher Scientific), as per manufacturer’s instructions.

### Flow cytometry

Cells were scraped and resuspended in FACS buffer (PBS + 2% FBS). Then cells were resuspended in normal rat serum (Fc block) for 25 min at 4°C. Live cells were stained with LIVE/DEAD^™^ Fixable Aqua Stain (Thermo Fisher Scientific). Next, cells were fixed with IC Fixation buffer (Thermo Fisher Scientific) for 30 min at room temperature and permeabilized with ice-cold methanol (Fisher Scientific) overnight at 4°C prior to antibody staining. Samples were stained with anti-p-p65-Alexa Fluor647 (clone 93H1; Cell Signaling), anti-p-S6-PerCP-eFlour710 (clone cupk43k; Thermo Fisher Scientific), and anti-IL-1b-APC-eFlour780 (clone NJTEN3; Thermo Fisher Scientific) antibodies for 25 min at 4°C protected from light. Following staining, the cells were centrifuged and resuspended in FACS buffer. Acquisition was performed with Attune NxT (Thermo Fisher Scientific). The Flow cytometry data were analyzed using FlowJo software (BD, Franklin Lakes, NJ).

### Real-time imaging of insulin signaling using fluorescent microscopy

L6 cells transfected with FoxO1 biosensor were seeded onto a 96-well plate for treatment. When cells reached 50–60% confluency, they were treated with conditioned media collected from THP-1 cells that were stimulated with LPS in the presence of control or copper leachates for 24 hours. After incubation time, all cells were starved in 0% FBS AMEM for 90 min, followed by treatment with 100 nM insulin. Images were taken using EVOS FL Auto 2 over a span of 30 min to observe fluorescent translocation. Nuclear fluorescence signal was traced from the nucleus to the cytosol in all treatments with number of cells being 10 for each treatment. Data quantitation was performed using Celleste software from Thermo Scientific.

### Statistical analysis

All statistical analysis was done using GraphPad software (Prism) using two-way analysis of variance (ANOVA) for comparison of multiple groups, or unpaired t-test (non-parametric Mann-Whitney test) for two groups. Following a significant main effect for interaction, post-hoc analyses (multiple comparison t-tests) were conducted to detect differences over time and between groups, with p-values as indicated in the figure legends.

## Results and discussion

### Copper-infused fabric attenuates inflammatory gene expression in human macrophages

**Fig 1:**
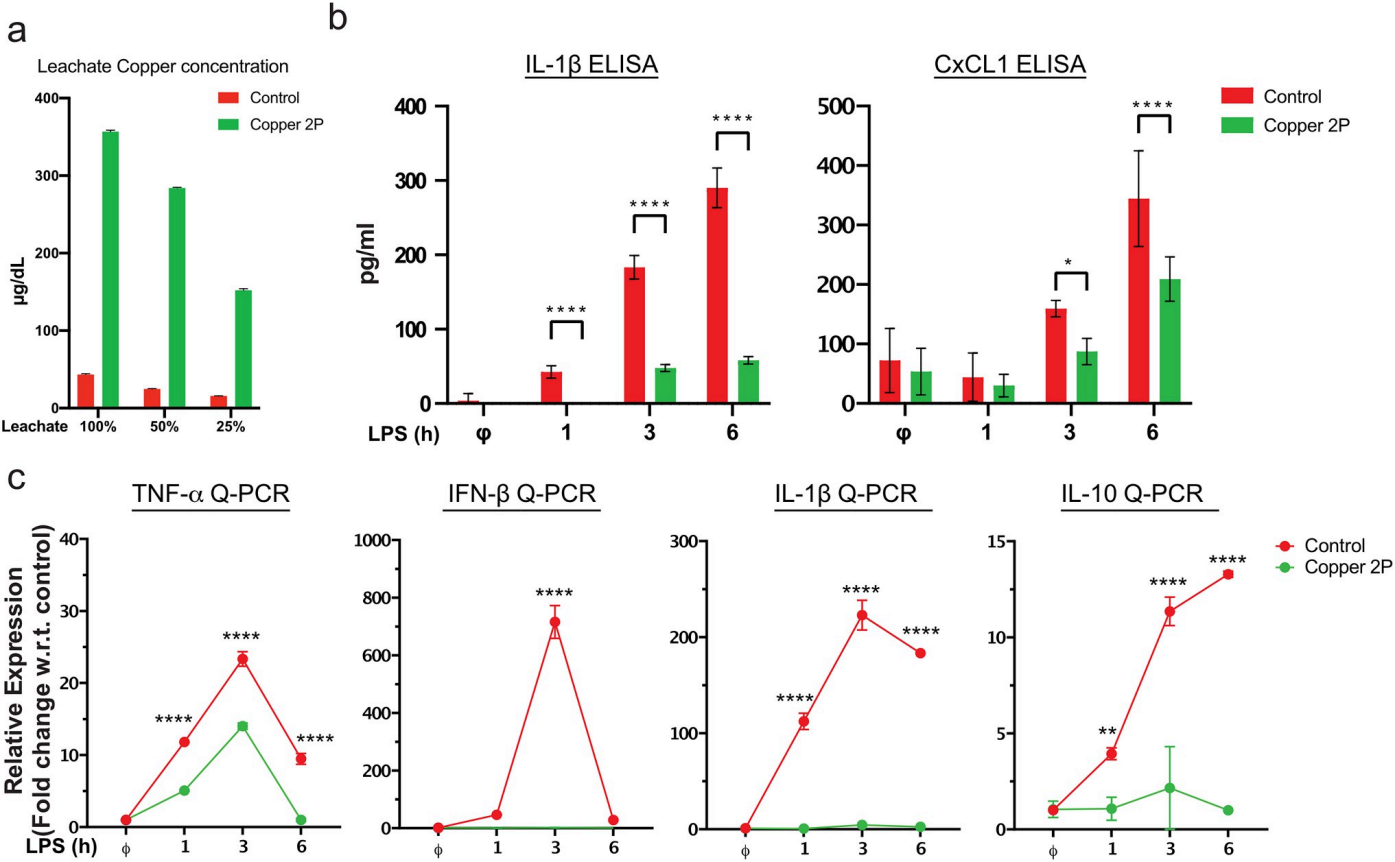
Copper-infused fabric dampen inflammatory gene expression. (**a**) copper assay showing the concentration of copper in undiluted leachate (100%), a 1:1 ratio of leachate to normal growth media (50%) or 1:3 ratio of leachate to normal growth media (25%). (**b, c**) THP-1 human monocytic cells were differentiated into macrophages following incubation with 100 nM PMA overnight. Cells were then cultured with 100% copper-infused fabric (Copper 2P) or non-copper fabric (control) leachates that were collected from pre-incubating the Copper 2P and control fabrics in media for 24 hours. Next, cells were stimulated with 100 ng/ml LPS for 1, 3, and 6 hours. Non-induced cells (ϕ) were only incubated with copper 2P and control leachates, and included as a negative control. (**b**) IL-1β and CxCL1 production were measured in supernatants of cultured cells by ELISA. (**c**) Gene expression of TNF, IFN-β, IL-1β and IL-10 was evaluated by real-time PCR (Q-PCR). GAPDH was used as a reference gene to normalize the data. Data from all experiments are representative of at least 3 independent experiments. * p<0.05; ** p<0.01; **** p<0.0001.

Macrophages are tissue resident immune cells that respond and initiate the inflammatory response to infections. Therefore, we used macrophages that were derived from the human THP-1 monocytic cell line (a.k.a. THP-1 derived macrophages) to examine the effect of copper infused fabrics on the initiation of inflammatory responses. THP-1 derived macrophages were incubated with media leachates from copper-infused fabric (Copper 2P) and matching fabrics that do not contain any copper (control) for 24 hours (Fig 1a) before being stimulated with LPS, a component of bacterial cell wall known to induce potent inflammatory responses, for various time points (1, 3, and 6 hours). Copper caused a significant reduction in LPS-induced inflammatory cytokine (IL-1β) and chemokine (CxCl1) production from THP-1 derived macrophages (Fig 1b). Similarly, inflammatory gene expression (TNF-α, IFN-β, and IL-1β) were significantly reduced in copper treated THP-1 derived macrophages (Fig 1c). This also included a reduction in gene expression of IL-10, an anti-inflammatory cytokine, which indicated that copper-infused fabrics were likely inhibiting a common upstream signaling component. Unsurprisingly, treatment of LPS-stimulated THP-1 cells with 20 μM copper salt (CuCl2), an amount equivalent to that present in 100% leachate, also led to reduced pro-inflammatory gene expression, as been previously reported [23] (S1 Fig).

**Fig 2:**
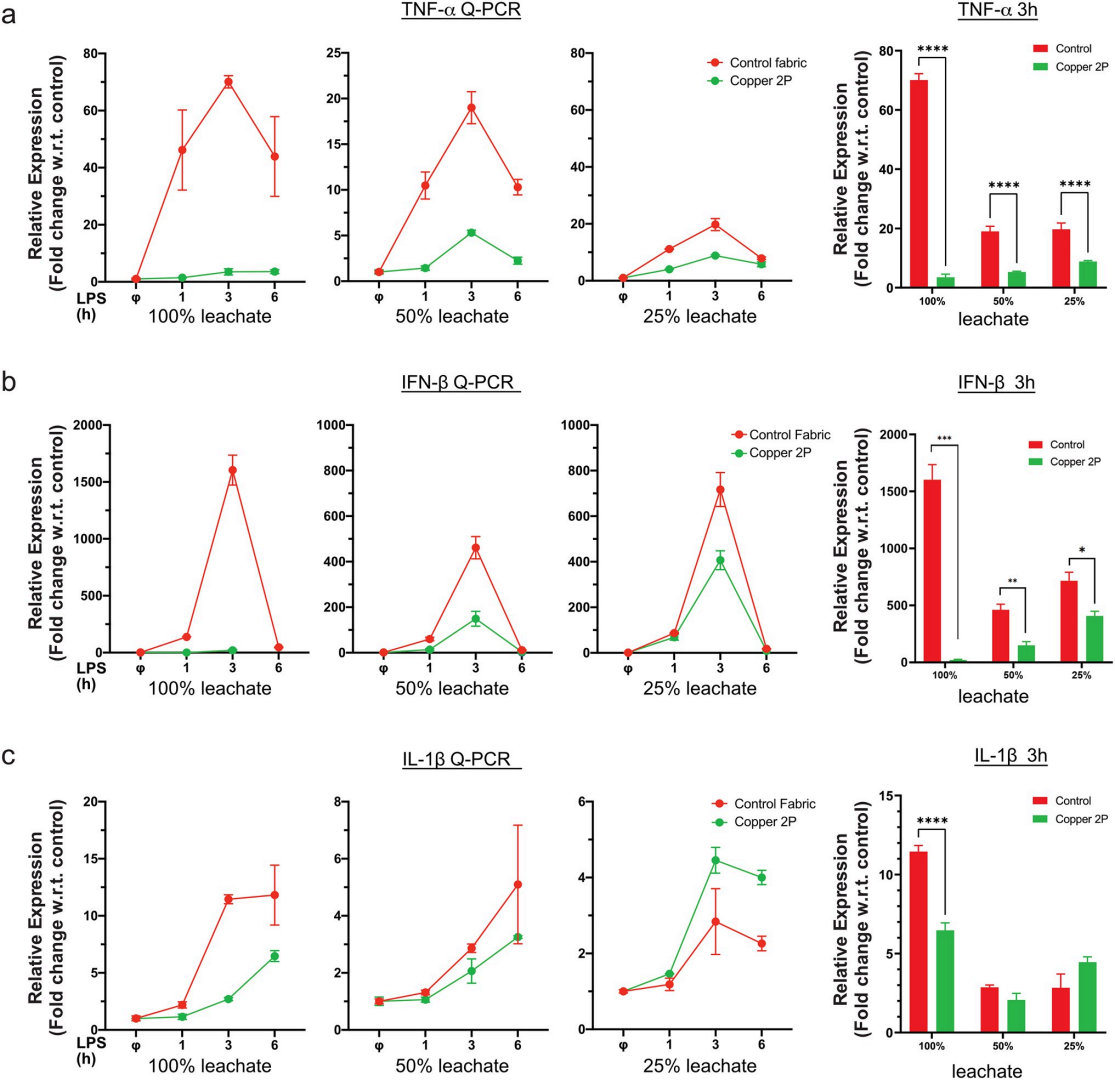
Dose-dependent effects of copper-infused fabric on inflammatory gene expression. PMA differentiated THP-1 cells were cultures in 100% leachate, 50% leachate or 25% leachate for 24 hours, as in Fig 1a, and then stimulated with 100 ng/ml LPS for the indicated times. Non-induced cells (ϕ) have been included as a negative control. Gene expression of (**a**) TNF-α, (**b**) IFN-β and (**c**) IL-1β was evaluated by real-time PCR (Q-PCR). GAPDH was used as a reference gene to normalize the data and plotted as a time course for each leachate concentration (left panels) or at the peak response (3h) to show bar graphs with all three concentrations (right panel). Data from all experiments are representative of at least 3 independent experiments. * p<0.05; ** p<0.01; *** p<0.001, **** p<0.0001.

Next, we examined whether this inhibitory effect is dose dependent. Exposure of THP-1 derived macrophages to media leachate that was increasingly diluted with regular growth media (Fig 1a) showed that the ability of copper-infused fabric to inhibit inflammatory gene expression persisted even when the leachates were diluted up to 4 times (Fig 2). We noted a dose dependent effect of copper-leachate mediated reduction in IFN-β expression, which may be due to the fact that IFN-β expression is under the control of a different transcription factor than TNF-α and IL-1β. Our data indicate that even at lower concentrations (~150 μg/dL), copper was still able to reduce inflammatory gene expression from LPS stimulated macrophages. Lower leachate concentrations may be needed to observe dose dependent effects on TNF-α and IL-1β.

**Fig 3:**
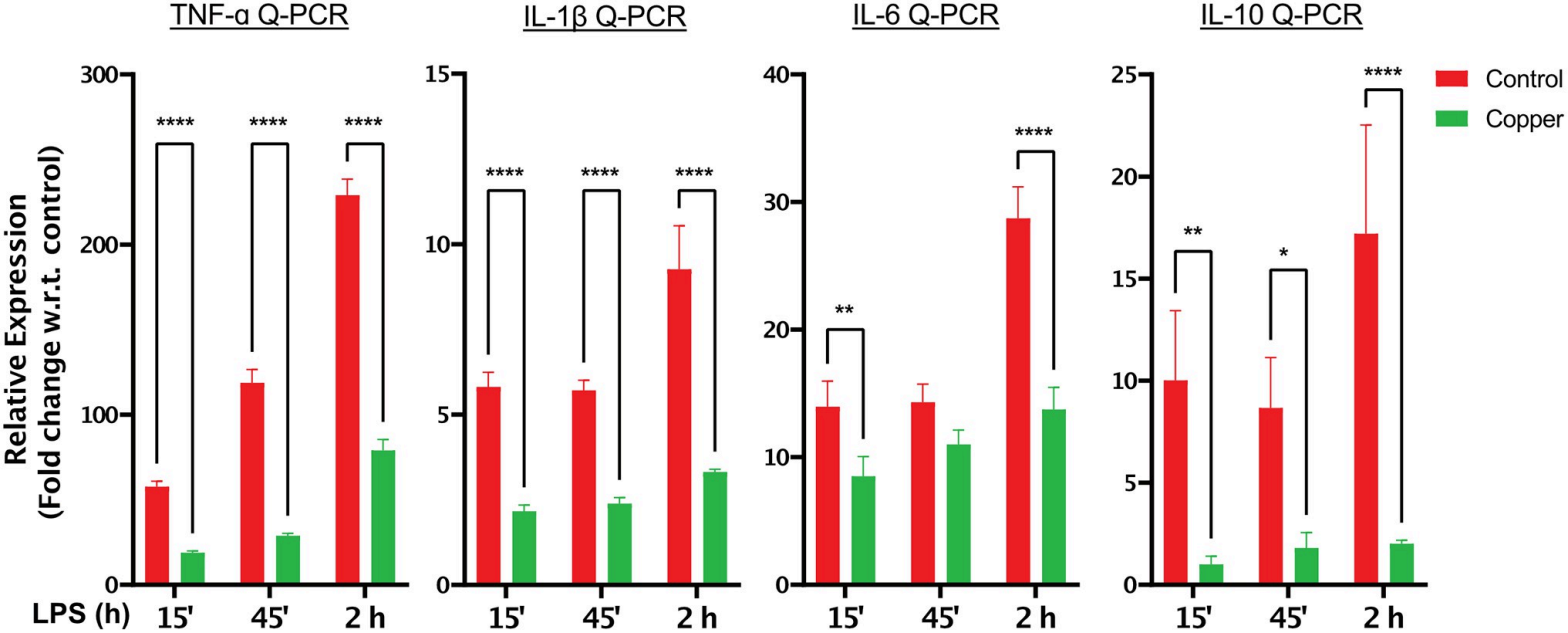
LPS driven inflammation can be attenuated following incubation with copper-infused fabric. PMA differentiated THP-1 cells were stimulated with 100 ng/ml LPS in normal growth media for 15 min before media replacement with leachates obtained from control or copper containing fabric. 100 ng/ml LPS was added to the leachates to maintain inflammatory cytokine stimulation. Cells were then incubated for an additional 15 min (15’), 45 min (45’) or 2 hours (2h). TNF-α, IL-1β, IL-6 and IL-10 gene expression was evaluated by real-time PCR (Q-PCR). GAPDH was used as a reference gene to normalize the data. Data from all experiments are representative of at least 3 independent experiments. * p<0.05; ** p<0.01; *** p<0.001, **** p<0.0001.

We then sought to determine whether copper can lower inflammation after inflammation has already been triggered. To this end, we stimulated THP-1 derived macrophages with LPS for 15 min, and then replaced the media with leachates from control fabric or Copper 2P and continued LPS stimulation for an additional 15, 45 or 120 min. Remarkably, copper leachates significantly decreased the production of TNF-α, IL-1β and IL-6 even when added after macrophages have been stimulated with LPS (Fig 3). The anti-inflammatory cytokine, IL-10, was similarly reduced in copper treated cells. Overall, these results indicate that copper can significantly attenuate activation and progression of inflammatory responses.

### Copper-infused fabric reduced NF-κB and IRF3 activation in LPS-stimulated macrophages

**Fig 4:**
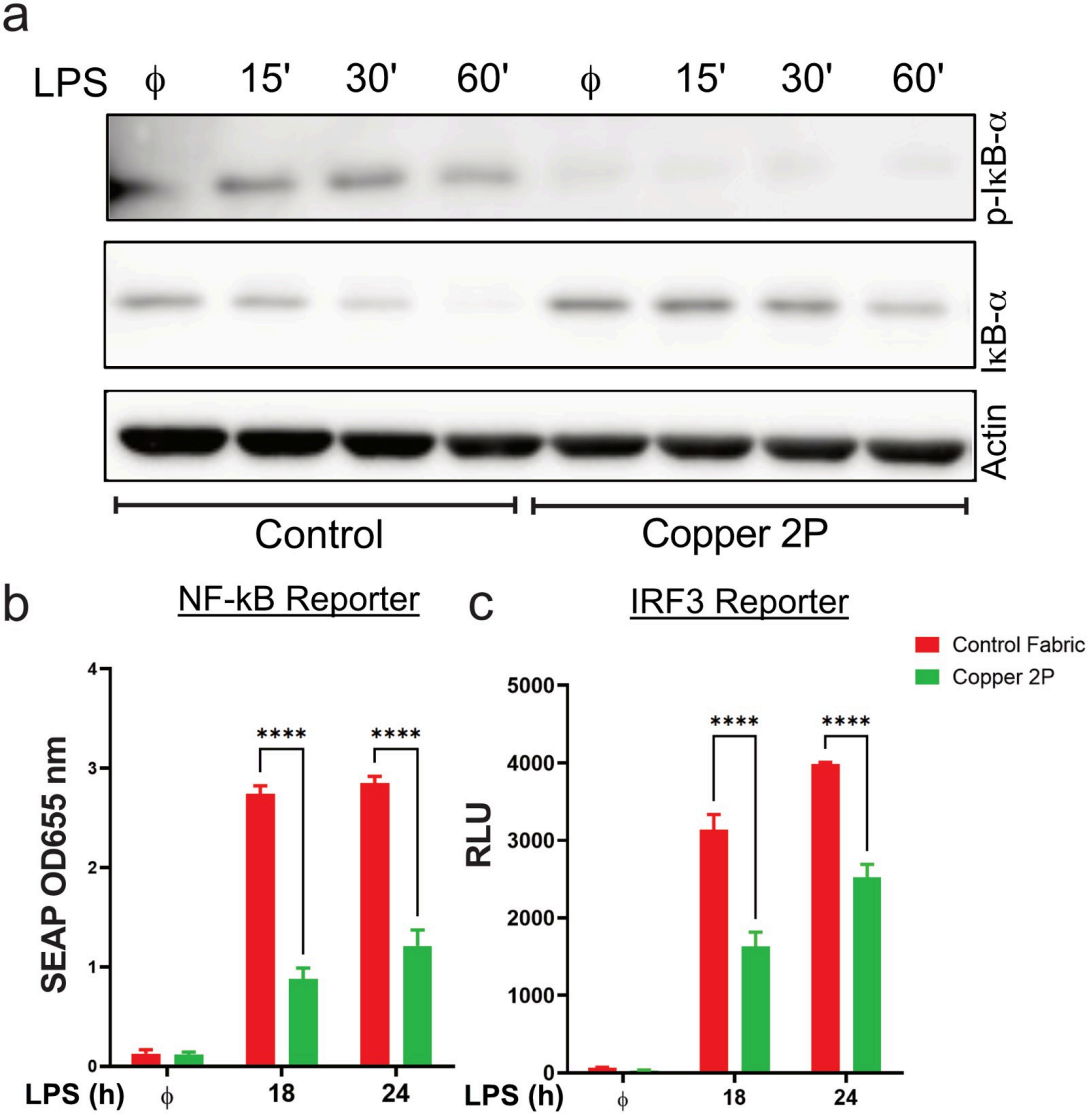
Copper-infused fabrics reduce LPS induced NF-κB and IRF3 activation. (**a**) PMA differentiated THP-1 cells were cultured in growth media that was pre-incubated with non-copper containing fabric (control fabric) or copper-infused fabric (Copper 2P) for 24 hours, then stimulated with 100 ng/ml LPS for the indicated times. Non-induced cells (ϕ) have been included as a negative control. Whole cell extracts were then prepared from these cells and immunoblotted for phosphor-specific IκB-α (p-IκB-α) or total IκB-α (IκB-α), and as a loading control, β-Actin. (**b, c**) The reporter mouse macrophage cell line RAW-Dual^™^ cells were cultured in growth media that was pre-incubated with non-copper containing fabric (control fabric) or copper-infused fabrics (Copper 2P) for 24 hours, then stimulated with 100 ng/ml LPS for the 18 or 24 hours. Non-induced cells (ϕ) have been included as a negative control. (**b**) NF-κB activation was determined using QUANTI-Blue^™^, a SEAP detection reagent, and by reading the optical density (OD) at 655 nm, and (**c**) IRF3 activation was determined by measuring the relative light units (RLUs) in a luminometer using QUANTI-Luc^™^, a Lucia luciferase detection reagent. Data from all experiments are representative of at least 3 independent experiments. * p<0.05; ** p<0.01; *** p<0.001, **** p<0.0001.

Gene expression of the pro-inflammatory cytokines, IL-1β, IL-6 and TNF-α, as well as that of the anti-inflammatory cytokine, IL-10, are induced when the transcription factor, NF-κB (typically a heterodimer of p65 and p50), is activated following the degradation of its inhibitor, IκB. p65 and p50 are then phosphorylated and translocate to the nucleus to drive expression of the aforementioned genes [7, 24]. On the other hand, IFN-β gene expression is driven by the transcription factor, IRF3, which like NF-κB, is activate by LPS stimulation and gets phosphorylated before forming a homodimer and translocating to the nucleus to drive gene expression [7]. Since we showed that copper-infused fabric can reduce gene expression of pro- and anti-inflammatory NF-κB induced genes as well as IRF3 induced genes, we tested whether these fabrics are affecting the activation of these upstream transcription factors. Indeed, NF-κB activation in LPS stimulated THP-1 derived macrophages was significantly lower in cells that were exposed to leachates from copper-infused fabric when compared to those from control fabric (Fig 4a). This was evident by a reduction in p-IκB levels and total I-κB degradation. A parallel set of studies was performed on a murine macrophage cell line, Raw 264.7, that reports the activation of both NF-κB and IRF3 transcription factors (Raw-Dual; Invivogen). Our data demonstrate that exposure to media leachates from copper fabric causes a significant reduction in both NF-κB and IRF3 reporter activities when Raw-Dual cells were stimulated with LPS (Fig 4b and 4c). These results indicate that copper-infused fabric can block inflammatory cytokine expression by inhibiting the activation of two key transcription factors, NF-κB and IRF3.

### Reduced inflammatory signaling in LPS-stimulated macrophages exposed to media leachates from copper-infused fabric

**Fig 5:**
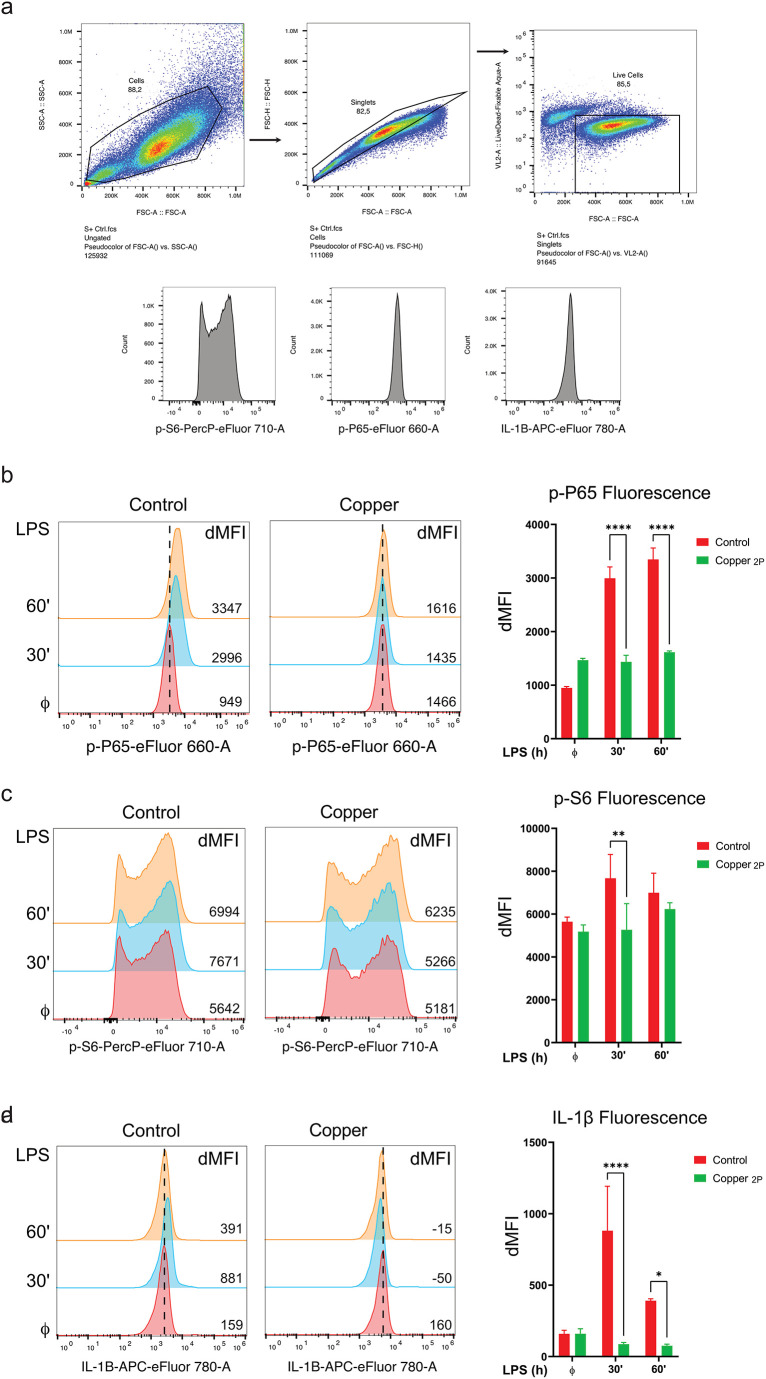
Copper-infused fabric reduce inflammatory signaling and proteins in LPS-stimulated macrophages. (**a**) Gating strategy for intracellular flow cytometry analysis performed on PMA differentiated THP-1 cells for measurements of phospho-P65 (p-P65), phospho-S6 (p-S6), and Interleukin-1 beta (IL-1β). (**b-d**) PMA differentiated THP-1 cells were cultured in growth media that was pre-incubated with non-copper containing fabric (control fabric) or copper-infused fabric (Copper) and stimulated with 100 ng/ml LPS for 30 or 60 min as indicated (30’ and 60’, respectively). Non-induced cells (ϕ) have been included as a negative control. Intracellular protein expression of (**b**) phospho-P65 (p-P65), (**c**) phospho-S6 (p-S6), and (**d**) Interleukin-1 beta (IL-1β) were measured by flow cytometry. The panels to the right are bar graphs plotting the dMFI, which is the mean fluorescence intensity (MFI) of each sample minus that of the fluorescence minus one (FMO) control. Data from all experiments are representative of at least 3 independent experiments. * p<0.05; ** p<0.01; *** p<0.001, **** p<0.0001.

In order to further elucidate the mechanism of copper-mediated anti-inflammatory effects, we investigated whether copper-infused fabric can alter the phosphorylation and subsequent activation of inflammatory signaling proteins in macrophages. These include IL-1β, the NF-κB subunit p65 as well as S6, a substrate of the mTOR signaling pathway which is activated to meet the high metabolic demand in the cell following LPS stimulation [25–27] (Fig 5a). Flow cytometric analysis of LPS-stimulated THP-1 derived macrophages showed that copper attenuated the phosphorylation of both p65 and S6 when compared to control fabric, as evident from the increase (i.e. rightward shift) in fluorescence intensity (MFI) of phospho-specific antibodies, indicated in the histograms (Fig 5b and 5c; left panels) and quantified in bar graphs (Fig 5b and 5c; right panel). Importantly, this was accompanied by inhibition of IL-1β cytokine production in cells exposed to media leachates from copper fabric (Fig 5d). Taken together, these results indicate that media leachates from copper-infused fabrics can potently block the phosphorylation of key inflammatory signaling proteins, which in turn, results in a significant reduction in production of inflammatory cytokines.

### Copper-infused fabric can reduce inflammation driven insulin resistance in skeletal muscle cells

**Fig 6:**
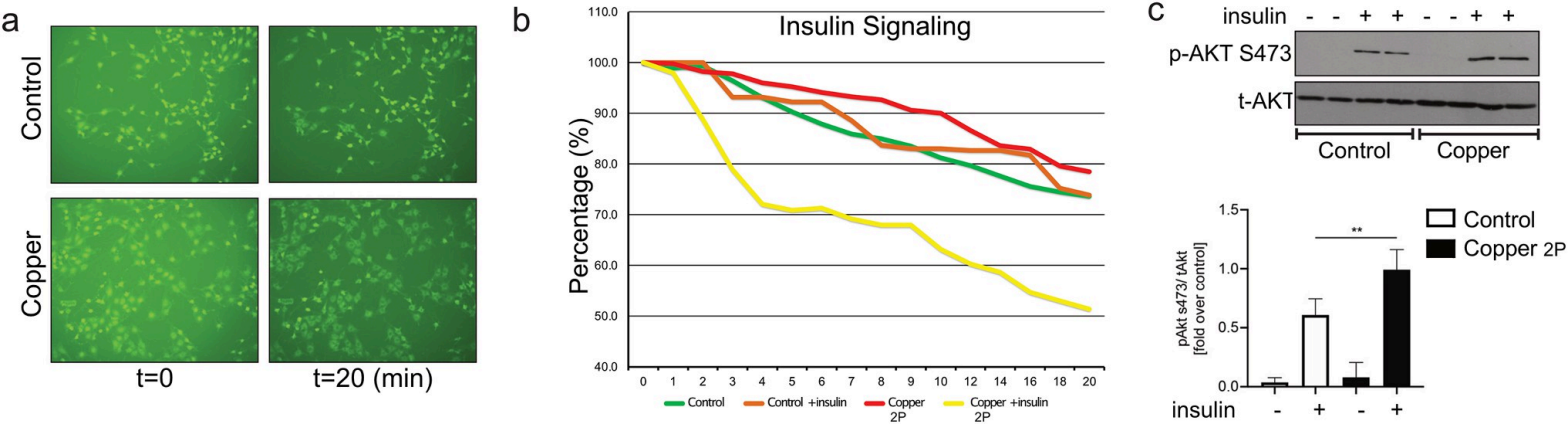
Copper rescues insulin sensitivity in L6 skeletal muscle cells. (**a**) Representative images of the biosensor-transfected L6 cells upon treatment with conditioned media obtained from LPS-stimulated THP-1 derived macrophages that were incubated leachate from non-copper containing fabric (control) or copper-infused fabric (Copper) for 20 min in the presence of 100 nM insulin. (**b**) Percentage of change in fluorescent signal observed in biosensor- transfected cells in control and copper treated media, as in (a), in the presence or absence of 100 nM insulin. The fluorescent intensity was measured manually for the duration of 20 min. (**c**) Representative Western blot images and quantification of phospho-specific Akt (pAkt s473) over total Akt (tAkt) after incubation in conditioned media as in (a) for 24 hours. Data from all experiments are representative of at least 3 independent experiments. **P < 0.01.

Chronic inflammation and pro-inflammatory cytokines can induce insulin resistance (reviewed in [28]), which, in turn, is linked to various diseases like obesity, type 2 diabetes, and heart failure [29]. A recent study showed that copper-infused fabrics reduced weight gain and improved glucose tolerance in mice on a high-fat high-cholesterol (HFHC) diet [30]. Therefore, we incubated rat skeletal muscle cells (L6) in conditioned media obtained from THP-1 derived macrophages that have been stimulated with LPS in leachates from Copper 2P or control fabric. To this end, we assessed insulin sensitivity in a skeletal muscle cell line, L6 cells, by two methods. We measured insulin-induced Akt phosphorylation by immunoblotting, the gold-standard approach to determine alterations in insulin signaling [31–33]. We also used L6 cells stably expressing an Akt biosensor, which relies on the translocation of fluorescently tagged FoxO1 from the nucleus to cytosol. FoxO1 is a direct downstream target of Akt kinase activity, and its nuclear extrusion is dependent on phosphorylation rendering the appearance of cytosolic FoxO1 as an indicator of Akt activity [33, 34]. Remarkably, copper containing conditioned media from LPS-stimulated macrophages showed a significant increase in insulin sensitivity in L6 cells (Fig 6). This is evident from enhanced insulin signaling (Fig 6a and 6b) and increase Akt phosphorylation (Fig 6c) in cells treated with copper containing conditioned media.

## Discussion

Exuberant inflammation, characterized by excessive production of pro-inflammatory cytokines, is the root cause for various human diseases, including type 2 diabetes, cardiovascular disease, and autoimmune diseases. Fine Cotton Factory Inc. (Toronto, ON, Canada) developed proprietary formulations to infuse copper ions and salts into fabrics to make clothes, bedding sheets, eye masks, compression socks and braces. While such products are being marketed as having anti-inflammatory effects based on anecdotes from customer testimonials, solid scientific evidence, and mechanistic insights into if and how copper-infused fabrics have anti-inflammatory effects are lacking. Results from this study provide evidence that leachates from copper-infused fabric can limit the activation of NF-κB and IRF3, two key transcription factors required for the induction of inflammatory cytokines. We show that cytokine gene expression and production is also reduced when human macrophage cell lines are exposed to media leachates from copper-infused fabric. Moreover, copper infused fabric can reduce inflammatory signaling induce by LPS stimulation. Consequently, copper can improve insulin sensitivity in skeletal muscle cells treated with conditioned media obtained from LPS stimulated macrophages. Metals, including copper and silver, can impose antimicrobial activities by being absorbed through the skin after leaching out from impregnated fabrics due to contact with biological fluids (e.g. saliva and sweat) [35–39]. Studies investigating mechanisms of metal infused fabrics on human cells generally relies on incubating the fabrics in a solution to allow the metals to leach out before exposing the cells to these leachates [40]. We used a similar approach to examine for the first time the anti-inflammatory effects of copper-infused fabrics on human macrophage cells. Since we cannot quantify the amount of copper that gets absorbed through the dermis, we diluted the leachates to show that lower leachate concentration continues to possess anti-inflammatory properties. Importantly, a previous study used the same copper-infused fabric and used them as bedding liners for mice to show that copper can attenuate insulin resistance in a diet-induced obesity model [30]. Although this indicates that copper from these fabrics may be absorbed through the skin and exert biological effects, additional in vivo studies are needed before drawing conclusions on whether copper-infused fabric provide anti-inflammatory benefits to consumers. Nevertheless, our findings provide novel evidence that demonstrate the anti-inflammatory effects of copper-infused fabric.

## Supporting information

S1 Fig Copper-infused fabric dampen inflammatory gene expression. (**a**) THP-1 human monocytic cells were differentiated into macrophages following incubation with 100 nM PMA overnight. Cells were then treated with 20 μM of copper(II) chloride dihydrate (CuCl2) or incubated with 100% leachate from copper-infused fabric (Copper 2P) or non-copper fabric (control) that were collected from pre-incubating the Copper 2P and control fabrics in media for 24 hours. Next, cells were stimulated with 100 ng/ml LPS for 1, 3, and 6 hours. Gene expression of TNF and IFN-β was evaluated by real-time PCR (Q-PCR). GAPDH was used as a reference gene to normalize the data. Data are representative of 3 independent experiments. (**b**) THP-1 derived macrophages were cultured with leachates from 100% copper-infused fabric (Copper 2P), Tommy Copper-infused fabric (commercial copper fabric) or non-copper fabric (control) and stimulated with 100 ng/ml LPS for 1, 3, and 6 hours, as in panel a. Non-induced cells (ϕ) were only incubated with copper 2P and control leachates and included as a negative control. Gene expression of TNF, IFN-β, IL-1β and IL-10 was evaluated by real-time PCR (Q-PCR). GAPDH was used as a reference gene to normalize the data. These data are the same ones presented in Fig 1c and are representative of at least 3 independent experiments. * p<0.05; ** p<0.01; **** p<0.0001.
(TIFF)

S1 Raw images (PDF)
