# Supplementary figures and images for: Copper infused fabric attenuates inflammation in macrophages
Source: PLoS One. 2023 Sep 15;18(9):e0287741. doi: 10.1371/journal.pone.0287741 (PMC10503751; doi:10.1371/journal.pone.0287741)

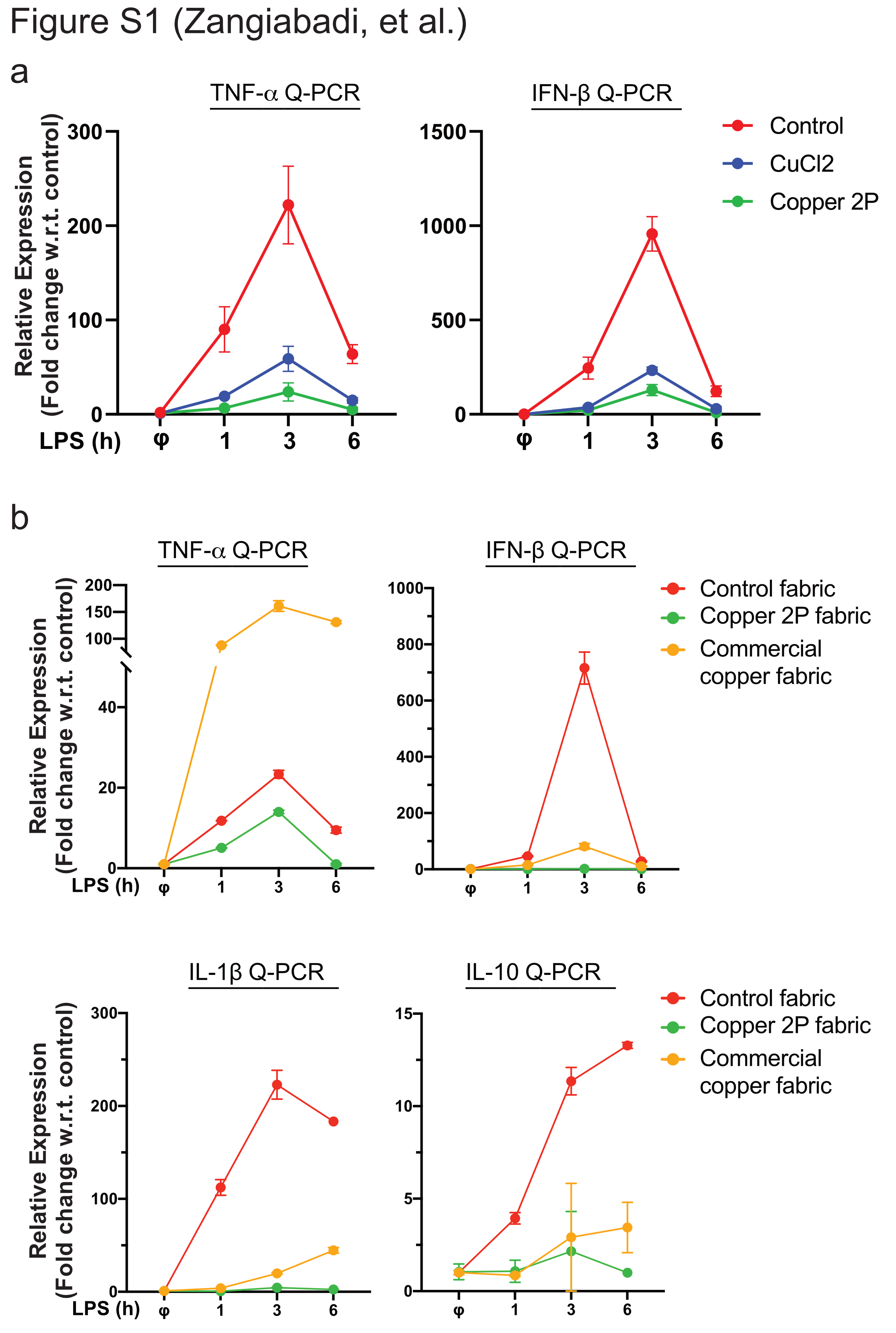

Supplement: S1 Fig — (a) THP-1 human monocytic cells were differentiated into macrophages following incubation with 100 nM PMA overnight. Cells were then treated with 20 μM of copper(II) chloride dihydrate (CuCl2) or incubated with 100% leachate from copper-infused fabric (Copper 2P) or non-copper fabric (control) that were collected from pre-incubating the Copper 2P and control fabrics in media for 24 hours. Next, cells were stimulated with 100 ng/ml LPS for 1, 3, and 6 hours. Gene expression of TNF and IFN-β was evaluated by real-time PCR (Q-PCR). GAPDH was used as a reference gene to normalize the data. Data are representative of 3 independent experiments. (b) THP-1 derived macrophages were cultured with leachates from 100% copper-infused fabric (Copper 2P), Tommy Copper-infused fabric (commercial copper fabric) or non-copper fabric (control) and stimulated with 100 ng/ml LPS for 1, 3, and 6 hours, as in panel a. Non-induced cells (ϕ) were only incubated with copper 2P and control leachates and included as a negative control. Gene expression of TNF, IFN-β, IL-1β and IL-10 was evaluated by real-time PCR (Q-PCR). GAPDH was used as a reference gene to normalize the data. These data are the same ones presented in Fig 1c and are representative of at least 3 independent experiments. * p<0.05; ** p<0.01; **** p<0.0001. (TIFF) [file pone.0287741.s001.tiff]
