# Supplementary material for: Copper infused fabric attenuates inflammation in macrophages
Source: PLoS One. 2023 Sep 15;18(9):e0287741. doi: 10.1371/journal.pone.0287741 (PMC10503751; doi:10.1371/journal.pone.0287741)

Uncropped blots for Figure 4a: Data detected using ChemiDoc MP imaging system (Bio-Rad).

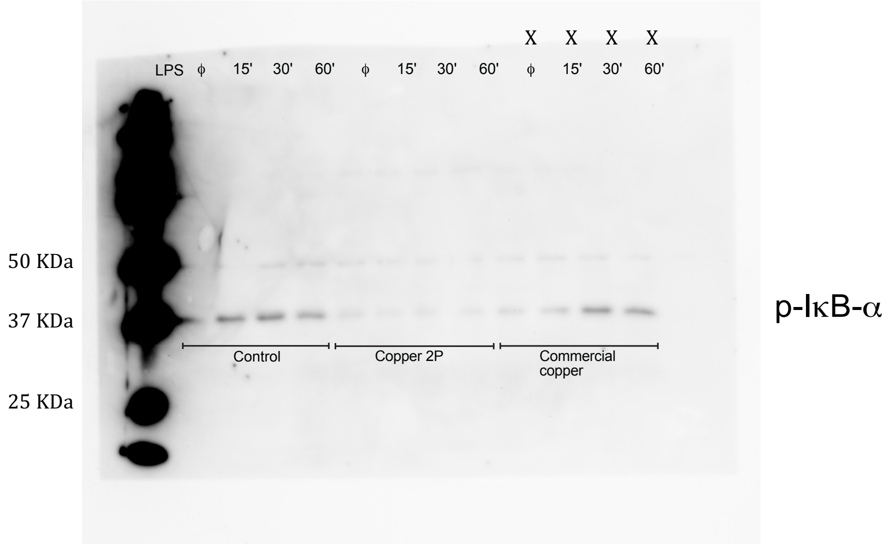

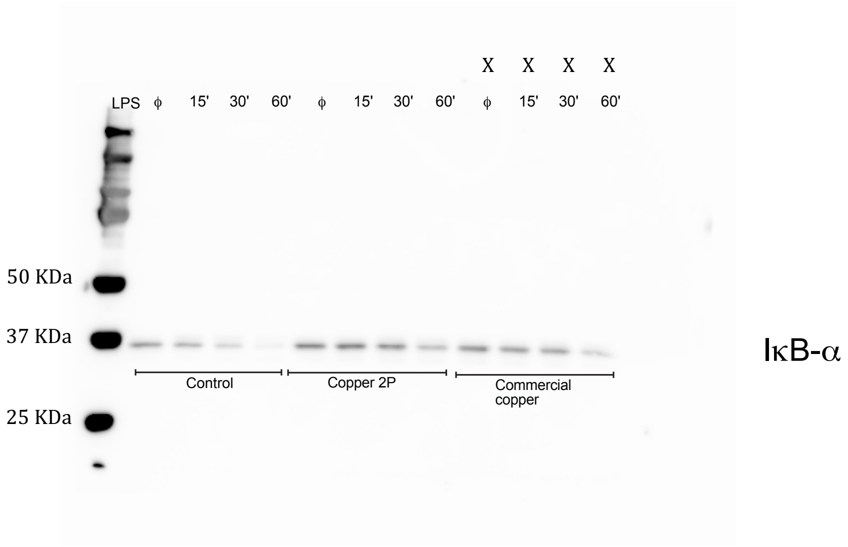

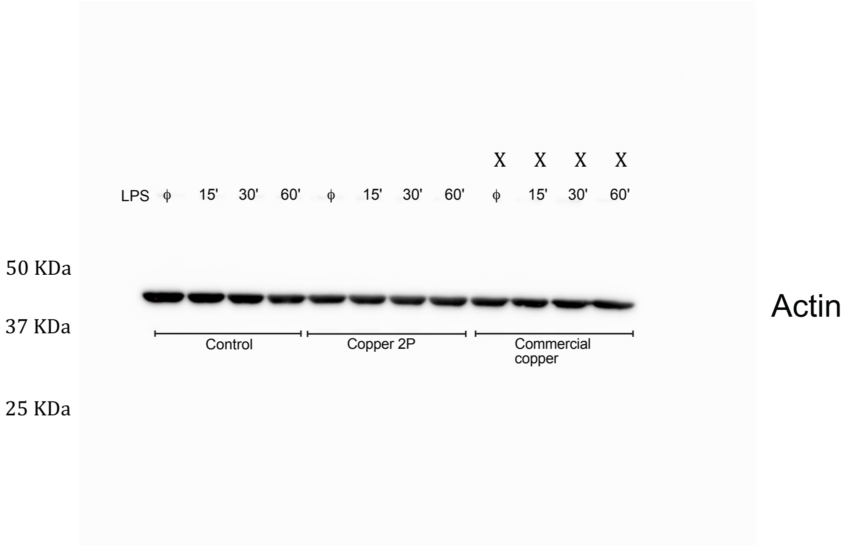

Uncropped blots for Figure 6c: Data detected on an x-ray film, developed, and then scanned.

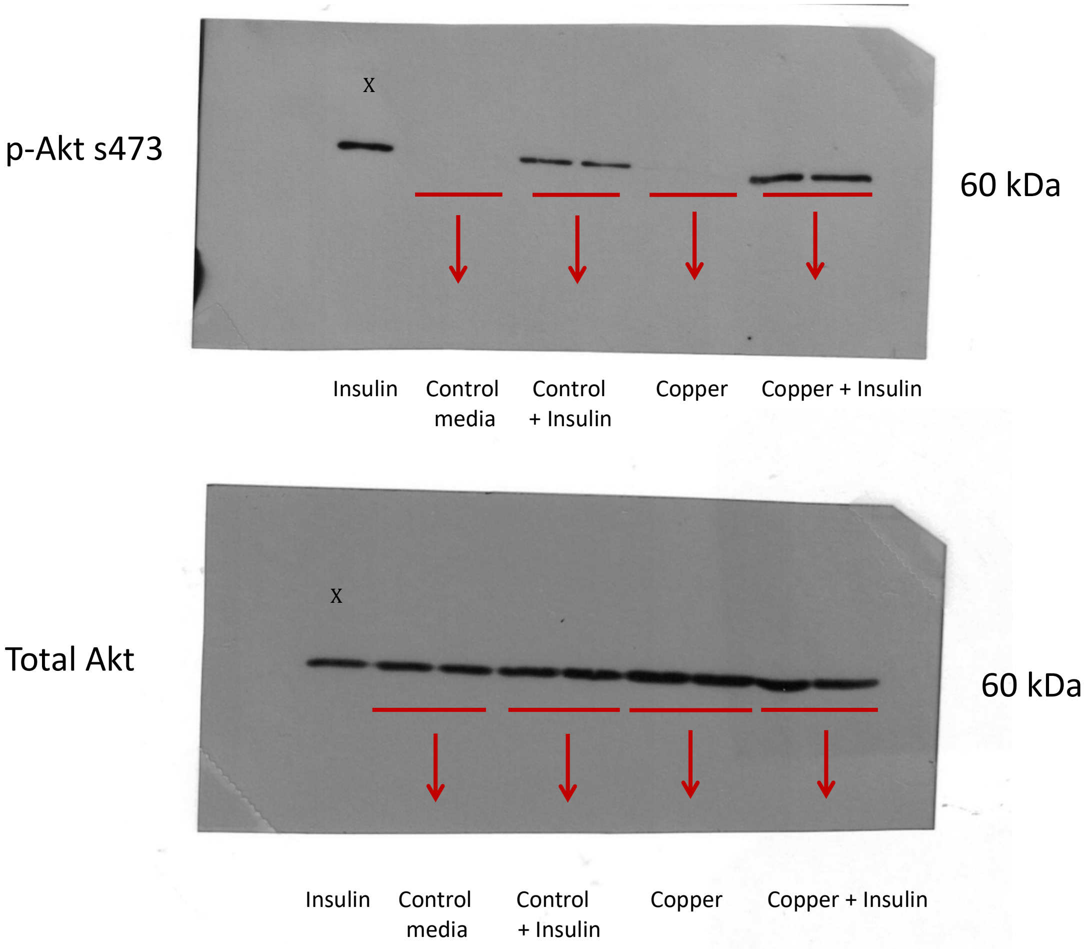

Supplement: S1 Raw images — (PDF) [file pone.0287741.s002.pdf]
